# The Nose—Its General Care and Management

**Published:** 1898-02

**Authors:** A. Bethune Patterson

**Affiliations:** Atlanta, Ga.


### THE NOSE—ITS GENERAL CARE AND MANAGEMENT.

BY A. BETHUNE PATTERSON, M. D., ATLANTA, GA.

As to the anatomy of the nose little need be said. The septum, or partition of the nose extends back to the posterior nares, and the pharynx or upper throat. The anterior portion of this upright partition is composed of cartilage, the posterior is of bone. On either side of the nasal cavities are three bony shelves, or projections, which stand out towards the septum, or middle partition, and are technically known as turbinate bodies. In normal conditions they do not extend far enough to touch the septum. They are designated as superior, middle, and inferior turbinates.

Opening into the nasal cavities are several canals, which communicate with important sinuses, or bony cellular chambers, found in the osseous structures bounding the nasal cavities.

Embedded in the mucous membrane are serous and mucous glands. The ducts, extending from them, open on the surface and between the ciliated epithelial cells; the latter are delicate structures forming the outer covering of the membrane.

The olfactory nerve filaments terminate in the mucous membrane covering the upper and superior part of the middle turbinate body; these are the nerves of smell. All odoriferous substances must reach the nerves by first being incorporated in the secretion covering the membrane.

The air, on passing through the nose, is warmed, moistened, and filtered of dust and other foreign particles; the hairs, found in the anterior nares, assist in this latter process.

I will now refer briefly to the four principal subdivisions of nasal catarrh. The word catarrh, to the professional mind, means inflammation of mucous membrane, never mind where found. To the unprofessional, this term has quite a different meaning; it is significant, not only of local trouble, but of constitutional infection, and considered incurable.

Acute catarrh is inflammation of the mucous membrane of the nose, or "cold in the head," which is often of a few days duration, while in many cases, it is characterized by recurrences; so the term "taking fresh cold" is quite common. This stage of catarrh deserves far more attention than it receives either from the physician or laity, from the fact, in my opinion, it is one of the principal causes of the other varieties.

Mothers and nurses should be taught the importance of early correcting or arresting catarrh in this stage. First by regulating diet, not overfeeding, proscribing candies, cakes, and the so-called rich foods, by not overclothing, and coddling the throat or neck, and keeping the apartments well ventilated.

Early in my practice, I learned that laxatives and tonics were the remedies for breaking up the habit of taking cold. I am now satisfied they are indispensable auxiliaries.

Recurrent attacks of acute catarrh lead to chronic hypertrophic, and atrophic varieties.

Cleansing the nose is an important adjunct in arresting the acute stage. Children should be instructed as early as possible in cleansing the nose with a weak solution of warm salt-water, or Dobell's solution, snuffed from the palm of the hand, or sprayed with an atomizer; this should become a part of the morning's toilet, and repeated several times during the day, accompanied with frequent use of handkerchief. When the acute stage has passed into the chronic and hypertrophic, cleansing becomes the most important home treatment.

The secretion from the glands and follicles becomes vitiated, thick, and tenacious, a medium in which germs of fermentation rapidly develop, becoming an additional source of irritation. When the mucous surface is kept clear of these, the tendency is to recovery.

In hypertrophic catarrh the mucous membrane becomes swollen and thickened, often blocking up the nose, obstructing free respiration and secretion, the latter often regurgitates into the upper pharynx, or throat, and either hawked and spat out or swallowed; in children it is more generally swallowed, also in adults while asleep. It is the general belief, that when the fermentative matter enters the stomach, it establishes fermentation, and dyspeptic symptoms, as well as blood troubles.

Nasal obstruction leads to mouth-breathing, which is a pernicious habit, often setting up pharyngeal, laryngeal, and bronchial irritation, besides the impairment of the voice, which must necessarily follow. Close and badly ventilated rooms, which are too often filled with tobacco smoke, intensifies every stage of catarrh and increases the suffering.

In atrophic catarrh the mucous membrane wastes away, becomes thin, the glands and follicles are destroyed, they no longer secrete mucous in quantities to moisten the nasal surfaces, it is greatly reduced it quickly dries, forming scabs, which become the home of putrefactive bacteria; the odors emitted are very offensive. The suffering is intensified by patches of ulceration, which underlie the scabs. The sense of smell is impaired and frequently destroyed. Atrophic catarrh is incurable; all that treatment can do, is to ameliorate the suffering, and make patients comfortable, which means daily treatment by washes and sprays of a cleansing character, as well as remedies which improve the general health, for constitutional tendencies play an important part in atrophic catarrh.

The frontal, sphenoidal, and maxillary sinuses frequently become complicated by an extension of catarrhal inflammation from the nasal cavities, and require treatment, which often proves unsatisfactory.

Nasal catarrh becomes the starting-point for pharyngeal, laryngeal, and bronchial catarrh.

The eye is often infected from the nose, the germs are transmitted through the lachrymal ducts; inflammatory and ulcerative affections of the conjunctiva and cornea are rendered more hazardous by this source of infection.

The eustachian tubes, which lead from the middle ear, open into the upper pharynx, in close proximity to the posterior nares. Nasal catarrh readily extends into the throat and through the tubes into the middle ear, and culminates in serious ear trouble.

I wish to urge the point of arresting catarrh in the acute stage. My co-workers will bear me out in saying, that only a very small percentage of acute catarrh in children is brought for treatment. It becomes the duty of practitioners to impress upon those who have the care of children the importance of early and prompt treatment.

Picking the nose with the finger is a habit not confined to the nursery, but it is quite common in adults. It invariably leads to superficial ulcerations, on the septum, just inside the vestibule; these ulcers often become the starting-point to perforations, and frequently the entire destruction of the cartilaginous portion of the septum. The scabs or dried secretion should not be removed by the finger, but should first be moistened and loosened by warm alkaline lotions snuffed up the nose, or sprayed; then blown out. Boracic acid ointment applied to the inner nose is very soothing.

I can not be far from wrong in stating that prophylaxis in nose and throat catarrh should command our attention, if for no other purpose than the prevention of consumption, which is often contracted from the air, through abraded and nude mucous surfaces of the respiratory organs.

